# *XAGE-1*基因在肺癌组织中的表达及其与临床特点的关系

**DOI:** 10.3779/j.issn.1009-3419.2010.08.07

**Published:** 2010-08-20

**Authors:** 群 赖, 立堂 张, 彦 孙

**Affiliations:** 277100 枣庄，山东省枣庄矿业集团中心医院心胸外科 Department of Thoracic Surgery, Central Hospital of Coal Group of Zaozhuang, Zaozhuang 277011, China

**Keywords:** *XAGE-1*基因, CT抗原, 肺肿瘤, 临床特点, *XAGE-1* gene, CT antigen, Lung neoplasms, Clinical characteristics

## Abstract

**背景与目的:**

研究证实XAGE-1作为一种癌胚抗原在多种肿瘤中高表达。本研究旨在探讨*XAGE-1*基因在肺癌组织中的表达水平及其与临床特点的关系。

**方法:**

肺癌患者85例，提取肺癌组织和癌旁组织总RNA，巢式PCR反应扩增*XAGE-1*四种剪接体基因，分析基因表达情况及其与临床特点的关系。

**结果:**

32.94%（28/85）肺癌患者癌组织阳性表达*XAGE-1*基因，在各种类型肺癌中，59.46%（22/37）的腺癌与21.74%（5/23）鳞癌患者癌组织阳性表达*XAGE-1*基因；*XAGE-1b*基因的表达与肺癌病理类型有相关性（*P* < 0.05），腺癌阳性表达率明显高于鳞癌，而与肺癌的性别、年龄及临床分期无相关性（*P* > 0.05）。

**结论:**

*XAGE-1*基因在肺癌尤其腺癌组织中高表达，该基因可以作为免疫治疗的靶基因。

## Abstract (en)

**Background and objective:**

XAGE-1 is a cancer-testis (CT) antigen which was demonstrated to be expressed at a significant frequency and to be immunogenic in some tumors. The aim of this study is to explore the association between *XAGE-1* gene expression and the clinical characteristics of lung cancer.

**Methods:**

Tumor tissue and adjacent lung tissue samples from 85 patients were screened for expression of the four XAGE-1 transcript variants by nest PCR. The correlations between *XAGE-1b* gene expression and several clinical characteristics were analyzed.

**Results:**

32.94% (28/85) of lung cancer samples were positive for *XAGE-1* gene. 59.46% (22/37) of the adencarcinoma samples and 21.74% (5/23) of the squamous cell carcinoma samples were positive for one of the four XAGE-1 transcript variants. The frequent of *XAGE-1b* gene in adencarcinoma was much higher than that in squamous cell carcinoma. There were not any important correlation between *XAGE-1b* gene expression and clinical characteristics, such as gender, age and clinical stage.

**Conclusion:**

*XAGE-1* gene is highly expressed in lung adenocarcinoma and XAGE-1 may be a promising immunotherapeutic target for lung cancer.

CT抗原（cancer-testis antigen）是一类在除睾丸、胎盘等以外的正常组织中几乎不表达的抗原，但可在多种肿瘤组织中表达，目前包括MAGE、BAGE、GAGE、NYESO-1、SSX等44种家族成员的CT抗原被发现^[1]^。通过RTPCR及免疫组化方法在肺癌、肝癌、卵巢肿瘤及前列腺癌等多种肿瘤中均表达CT抗原，同时研究^[1]^证实CT抗原可在肿瘤患者体内诱导特异性的细胞免疫及体液免疫应答。XAGE-1是1999年通过生物信息学技术发现的一种新的CT抗原，属于GAGE/PAGE家族，*XAGE-1*基因定位于Xp11.21-Xp11.22，包括*XAGE-1a*、*XAGE-1b*、*XAGE-1c*、*XAGE-1d*四种剪切体，利用RT-PCR及Northern blot发现*XAGE-1*基因在Ewing’s肉瘤、恶性黑色素瘤、前列腺癌、肝癌等多种肿瘤中表达^[2, 3]^。本实验研究*XAGE-1*四种不同剪接体在肺癌组织中表达情况并分析其与临床特点的关系，为XAGE-1作为肺癌免疫治疗靶点提供理论依据。

## 1 材料与方法

### 1.1 研究对象

随机选取2008年1月-2009年3月在我院胸外科住院手术患者85例（男性60例、女性25例，平均年龄56.5岁）; 腺癌37例，鳞癌23例，大细胞癌15例，小细胞肺癌10例，所有病例均经过病理证实，患者签署知情同意书。手术过程中，肺叶或全肺切除后，立即切取肿瘤组织及距肿瘤组织 > 5 cm的癌旁组织各3块，每块约0.5 g，置入无菌、去RNA酶的冻存管，立即投入液氮中，后转入-80 ℃冰箱保存。

### 1.2 方法

#### 1.2.1 组织细胞RNA提取

取约0.2 g肺癌组织及癌旁组织于液氮中研磨，加入Trizol试剂，提取总RNA，Trizol试剂购自美国Invitrogen公司，严格按照试剂说明书进行提取。所得RNA用DEPC处理过的水溶解，-80 ℃保存备用。取少量RNA在紫外分光光度计260 nm和280 nm下确认浓度和纯度。

#### 1.2.2 肺癌细胞系A549培养与RNA提取

用含10%小牛血清的RPMI-1640培养A549细胞，收集总数为5×10^6^个细胞利用Trizol试剂进行RNA提取，DEPC处理过的水溶解后-80 ℃保存备用。

#### 1.2.3 巢式PCR

采用cDNA第一链合成试剂盒进行合成，该试剂盒购自加拿大Fermentas公司，严格按照试剂说明书进行操作，所得cDNA在-20 ℃保存。根据文献^[2]^的方法略有改动进行巢式PCR扩增，在50 μL PCR反应体系中包括0.4 μmol/L引物、1.5 mmol/L MgCl_2_、200 mmol/L dNTP及2.5 U GoldTaq酶，第一轮PCR模板为4.0 μL cDNA，选用外侧引物进行扩增，反应条件如下：95 ℃预变性5 min，94 ℃、30 s，60 ℃、45 s，72 ℃、60 s，35个循环，72 ℃延伸7 min; 从第一轮PCR产物中取出1.0 μL作为第二轮PCR模板，反应条件为：95 ℃预变性5 min，94 ℃、30 s，58 ℃、30 s，72 ℃、30 s，30个循环，72 ℃延伸7 min; 同时以GAPDH（PCR产物为138 bp）为内参考基因同时进行扩增，PCR产物以2%琼脂糖凝胶进行电泳观察结果。

#### 1.2.4 PCR产物测序分析

每个阳性条带所对应的PCR产物进行测序，测序得到的序列利用BLAST程序在GenBank上进行序列分析。

### 1.3 统计学分析

采用SPSS 10.0统计软件对数据进行统计分析，利用*Fisher’s*精确概率法及*Pearson’s chi square*检验进行*XAGE-1b*基因表达与临床及病理特点相关性分析，以*P* < 0.05为有统计学差异。

## 2 结果

### 2.1 *XAGE-1*基因在肺癌组织中表达情况

收集85例肺癌组织和癌旁组织，提取RNA后利用巢式PCR检测*XAGE-1*四种不同剪接体基因的表达情况，结果发现部分肺癌组织表达*XAGE-1b*、*XAGE-1d*基因，而不表达*XAGE-1a*、*XAGE-1c*基因，A549细胞系表达*XAGE-1b*基因（图 1），PCR产物经测序后与GenBank进行序列比对后证实为目的基因，癌旁组织中均不表达*XAGE-1*基因。在各种类型肺癌中，59.46%（22/37）的腺癌组织表达*XAGE-1*基因（*XAGE-1a* 0/37; *XAGE-1b* 17/37; *XAGE-1c* 0/37; *XAGE-1d* 6/37），其中1例腺癌组织同时表达*XAGE-1b*与*XAGE-1d*基因。21.74%（5/23）鳞癌组织表达*XAGE-1*基因（*XAGE-1a* 0/23; *XAGE-1b* 4/23; *XAGE-1c* 0/23; *XAGE-1d* 1/23），15例大细胞癌组织中仅检测到2例癌组织表达*XAGE-1b*基因，而10例小细胞癌组织中均未检测到*XAGE-1*基因表达。

**1:**
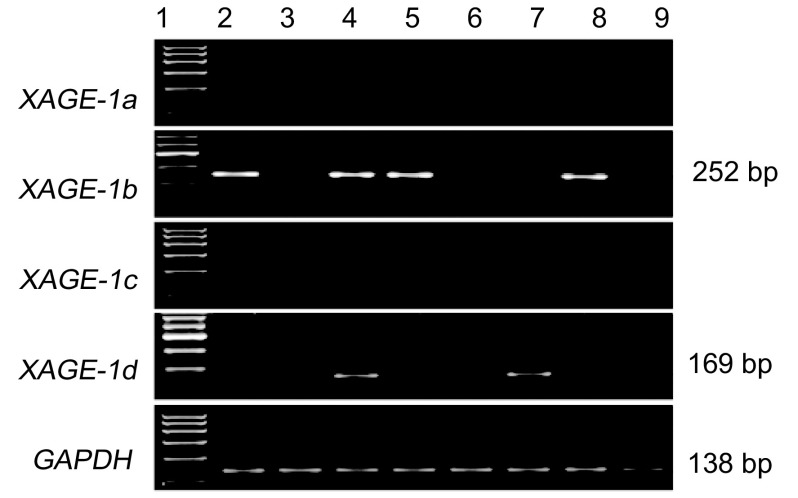
*XAGE-1*基因在肺癌组织及癌旁组织中的表达。1：10 0 bp DNAladder; 2：A549细胞系; 3：癌旁组织; 4、5：腺癌组织; 6、7：鳞癌组织; 8：大细胞肺癌组织; 9：小细胞肺癌组织。XAGE-1b、XAGE-1d和GAPDH的PCR产物大小分别为252 bp、169 bp和138 bp。 Expression of *XAGE-1* gene in lung tumor tissues and adjacent tissues. 1: 100 bp DNA ladder; 2: A549 cell line; 3: tumor adjacent tissue; 4, 5: adencarcinoma; 6, 7: squamous cell carcinoma; 8: large cell lung cancer; 9: small cell lung cancer. The sizes of PCR products are 252 bp, 169 bp and 138 bp for XAGE-1b, XAGE-1d and GAPDH, respectively.

### 2.2 *XAGE-1b*基因表达与腺癌、鳞癌临床特点的关系

目前研究认为XAGE-1b具有较高的免疫原性和较高的表达率，我们分析了37例腺癌与23例鳞癌组织标本*XAGE-1b*基因的表达与性别、年龄、病理类型及临床分期的关系（表 1）。统计结果表明*XAGE-1b*基因的表达与肺癌病理类型有相关性（*P* < 0.05），腺癌阳性表达率明显高于鳞癌，而与肺癌的性别、年龄及临床分期无相关性（*P* > 0.05）。由于对病例生存期随访时间较短，故有待于对病例进行继续随访，以便分析*XAGE-1b*基因的表达与患者生存期之间的关系。

**1:** *XAGE-1b*基因表达与腺癌、鳞癌患者临床特点的关系 The correlation between *XAGE-1b* gene expression and several clinical characteristics of adencarcinoma and squamous cell carcinoma

	*XAGE-1b* (+)	*XAGE-1b* (-)	Total	*P*
Gender				0.559 9
Male	16	26	42	
Female	5	13	18	
Age (year)				0.573 3
≥60	15	24	39	
< 60	6	15	21	
Histology				0.0289
Adencarcinoma	17	20	37	
Squamous cell carcinoma	4	19	23	
Clinical stage				0.1659
Ⅰ	5	17	22	
Ⅱ-Ⅲ	16	22	38	

## 3 讨论

肺癌是最常见的恶性肿瘤之一，其中非小细胞肺癌占70%-80%，目前手术及化疗效果特别是晚期患者效果不佳，免疫治疗是一种新的治疗方法，而免疫治疗的基础就是必须寻找特异性表达的肿瘤抗原。研究证实CT抗原具有较高的免疫原性，可以诱发机体的肿瘤特异性的细胞免疫和/或体液免疫杀伤肿瘤细胞，同时CT抗原仅在睾丸、胎盘等正常组织中表达，但在多种肿瘤组织中高表达，具有较高的肿瘤特异性，因此，CT抗原是肿瘤免疫治疗特别是肿瘤疫苗治疗的理想靶抗原，以NY-ESO-1、MAGE-A、SSX抗原为基础的肿瘤疫苗已进入临床试验阶段^[4-6]^。研究^[7]^发现在非小细胞肺癌组织中也表达MAGE-1、MAGE-2、MAGE-3、MAGE-12、NY-ESO-1等多种CT抗原。

XAGE-1是一种在多种肿瘤组织中表达的CT抗原，通过RT-PCR及Northern blot研究发现*XAGE-1b*基因在PC3（前列腺细胞系）、MDA-MB-231（肺癌细胞系）、OVCAR（卵巢细胞系）、FEM-X（黑色素瘤细胞系）、HUT102（T细胞淋巴瘤细胞系）、U937（组织细胞性淋巴瘤细胞系）、A-172（神经胶质细胞瘤细胞系）均有表达，同时有临床研究发现*XAGE-1b*基因在Ewing’s肉瘤、恶性黑色素瘤、前列腺癌、肝癌等多种肿瘤组织中表达，在前列腺癌和黑色素瘤的研究中还发现*XAGE-1b*基因表达与肿瘤的临床分期有关^[2, 3]^。本研究采用巢式PCR技术检测*XAGE-1*四种不同剪接体在肺癌中表达情况，结果显示32.94%（28/85）肺癌患者癌组织阳性表达*XAGE-1*基因，其中27.06%（23/85）肺癌患者癌组织阳性表达*XAGE-1b*基因，与国外报道的30.61%（15/49）肺癌患者癌组织阳性表达*XAGE-1b*基因略有差异，本研究还检测了肺癌组织中*XAGE-1d*基因表达情况，研究发现8.24%（7/85）患者癌组织阳性表达*XAGE-1d*基因，低于Nakagawa等^[8]^报道的表达阳性率（12.24%, 6/49），可能与病例数及病理类型组成不同有关。本研究证实XAGE-1b具有较强的免疫原性和较高的表达率，本实验重点分析了*XAGE-1b*基因的表达与临床特点的关系，结果发现*XAGE-1b*基因的表达与肺癌病理类型有相关性（*P* < 0.05），腺癌阳性表达明显高于鳞癌，与肺癌的性别、年龄及临床分期无相关性（*P* > 0.05），提示在肺腺癌中可以优先选择该基因作为瘤苗及免疫治疗靶点。Shimono等^[9]^鉴定出HLA-DRB1^*^0410限制的XAGE-1b_37-48_多肽可以产生XAGE-1b特异性的CD4^+^T细胞，Kikuchi等^[10]^已经报道患者在手术后XAGE-1b可以诱导CD8^+^T细胞抗微小残留病变从而延长患者的存活时间，从而进一步为XAGE-1作为肿瘤治疗的靶点提供了理论基础。

综上所述，本实验研究发现*XAGE-1*基因在部分肺癌组织阳性表达，*XAGE-1b*基因表达与肺癌病理类型有关。但目前XAGE-1在肿瘤细胞中发挥的生物学功能仍然未知，下一步应进一步对XAGE-1在肿瘤发生发展中的生物学作用进行深入研究，以便为XAGE-1作为免疫治疗靶点提供理论依据。
